# Comparison of asynchronous online versus in-person library instruction methods for teaching literature searching to graduate students

**DOI:** 10.29173/jchla29792

**Published:** 2024-12-01

**Authors:** Sandra McKeown, Angélique Roy, Wilma M. Hopman

**Affiliations:** 1 Health Sciences Librarian, Bracken Health Sciences Library, Queen’s University, Kingston, ON; 2 Health Sciences Librarian, Bracken Health Sciences Library, Queen’s University, Kingston, ON; 3 Research Methodologist and Biostatistician, KGH Research Institute; Adjunct Faculty, Department of Public Health Sciences, Queen’s University, Kingston, ON

## Abstract

**Introduction:**

The objectives of this comparative study were 1) to compare the effectiveness of in-person classroom instruction versus an online video for teaching literature searching skills to graduate students, and 2) to evaluate their perceptions of the instruction methods received and their instruction preferences for learning literature searching skills.

**Methods:**

Students enrolled in a translational medicine graduate course in Fall 2022 were invited to participate in the study. Participants were randomly assigned to a control group (receiving in-person instruction) or to an intervention group (receiving a link to a narrated PowerPoint video). Using Qualtrics online survey tool, participants completed one pre-test and two post-tests to assess learning and retention, and a survey to evaluate their perceptions and attitudes.

**Results:**

12 out of 17 students participated. Both instruction methods were effective for delivering this information literacy content. The in-person group improved more than the online video group from pre- to post-test 1 and from pre- to post-test 2, but the difference was not statistically significant. However, the online video group rated the pace, perceived effectiveness, and clarity of library instruction, and their confidence to perform specific search tasks, more favourably than the in-person group, although the difference was not significant. Participants valued being able to access library training on their own schedule most of all.

**Discussion:**

The sample size for this study was small, making it difficult for differences to attain statistical significance. Creating an online video to deliver this content ended up being very time-intensive compared to providing synchronous instruction.

## Introduction

The COVID-19 pandemic necessitated a shift from in-person instruction to various forms of online learning at colleges and universities in Canada and around the globe as buildings on campus closed their doors for extended periods of time. While computer-assisted instruction has been discussed considerably in relation to library instruction for the better part of five decades, the pandemic marked a quick turning point in the uptake of online instruction for many librarians. A survey distributed to academic librarians in 2021 found that 92.6% (n=225) changed to various forms of online instruction during COVID-19 [1]. Now that we have returned to campus and in-person instruction is once again possible, librarians may find it challenging to determine the most effective and desirable method for delivering information literacy content.

### Forms of online instruction

When considering types of online instruction, or e-learning, the two forms are synchronous and asynchronous. Synchronous instruction centres around active learning which can be defined as instructional methods that engage students in the learning process through activities, discussions, problem-solving, and reflection [2]. This approach is often contrasted with traditional lecturing where students passively receive information from the instructor and engage with the material presented through listening and notetaking. In synchronous online instruction, active learning includes collaboration and interactivity wherein students assume a more dynamic role in their engagement with the material. They are required to gather virtually at a designated time and interact with the instructor and possibly their peers in real-time. This method is facilitated by the instructor through the delivery of content and reinforcement activities. The instructor may easily pivot or change approaches based on immediate feedback from participants to adapt the lesson to students’ needs [3]. Popular software programs for synchronous online instruction include Zoom and Microsoft Teams [4].

While synchronous instruction offers real-time interaction, asynchronous instruction provides a different approach to active learning by placing the student at the centre of the educational experience offering flexibility as students can access and consume content on their own schedules and at their own pace [5]. This type of learning is self-directed without live interaction or input from the instructor and the learning object may act as a standalone resource which can be reviewed as little or as often as required by the learner or may be facilitated through integrated assignments or discussion boards [6]. Interactive modules using Guide-on-the-Side, LibGuides, or embedded within Learning Management Systems (LMS) are common approaches for asynchronous instruction.

The flipped classroom is an instructional strategy commonly used both online and in-person aimed at increasing student engagement by creating an environment conducive to students’ learning needs and preferences [7]. Readings or videos are provided to learners to complete independently prior to synchronous or asynchronous instruction that is centered on knowledge sharing through reflection activities, group discussions, instructor Q&A, work periods, or assessments. It offers students a form of autonomous and flexible learning by shifting course content outside of scheduled class time and favours active learning over passive by focusing on activities to engage with the new content. This instructional approach is also favourable with blended teaching combining asynchronous online learning with face-to-face instruction [8].

And while librarians may employ a broad range of teaching techniques and methods to engage students in online instruction, one common asynchronous format is online tutorials or modules, which often incorporate video recordings. When used in the blended or flipped classroom, these serve as an active learning method to combine the benefits of both synchronous and asynchronous learning [9,10]. Typically, pre-recorded videos are used for content delivery and may also be interactive with built-in quizzes or activities [10,11]. Once the asynchronous video is completed, a synchronous instructional portion, an in-person or online video call, incorporates the video content through collaborative discussions and activities to put learning into practice. Librarians may also employ the flipped classroom in an effort to contend with shortened class time dedicated to information literacy instruction. Recorded videos are tailored to the desired learning outcomes set by the librarian to fit within the curriculum or course objectives. These can be simpler screencasts to convey a point-of-need topic or divide complex topics into multiple sections or modules and embedded into websites, LibGuides, freely available on YouTube or embedded into an LMS [10,12].

### Background and objectives

Even before the pandemic, many academic libraries were utilizing online instruction, which can accommodate distant learners, give learners the ability to access content at the time of need, and offer flexibility and autonomy in learning. And although classroom instruction has its own benefits for learners and instructors, when the pandemic forced academic libraries to halt in-person instruction, many librarians transitioned to forms of online instruction. During this time, a health sciences librarian at Queen’s University in Kingston, Ontario, adapted an in-person library session to an online format for a group of graduate students. Students returning to campus in 2022 presented an opportunity to compare the effectiveness of traditional in-person classroom instruction with a form of asynchronous online instruction for teaching literature information literacy content.

The primary objective of this study was to compare the effectiveness of traditional in-person classroom instruction versus a narrated PowerPoint video for teaching literature searching skills to graduate students in the Translational Medicine Graduate Program. The secondary objective was to evaluate their perceptions of the instruction methods received and their preferences regarding instruction methods for learning literature searching skills.

## Methods

### Participants and setting

Graduate students enrolled in the Translational Medicine Graduate Program taking the course, *Research Success Skills*, in the fall term of 2022 were invited to participate in this study. The course instructor and teaching assistant for the course, as well as the graduate program director, approved the research proposal before ethics clearance was sought and received by the Queen's Research Ethics Board.

Since the inception of the Translational Medicine Graduate Program at Queen’s University in 2018, and the first offering of *Research Success Skills* that fall term, the liaison librarian for postgraduate medical education has provided three separate information literacy sessions for students. In the second week of classes, the librarian provides a 1.5-hour library orientation, introducing library services and resources. In the fourth week of classes, the librarian provides a session on literature searching in bibliographic databases. Following these sessions, each student completes a mandatory 30-minute research consultation with the liaison librarian sometime in the month of October to review their literature search approach for a specific research question (conducted online using Zoom because the library was temporarily closed for construction). Students receive feedback on their search approach during their individual consultation, but the librarian does not provide a formal evaluation or assign a mark for this work.

### Library session description

The information literacy content covered in this comparative study was about literature searching in bibliographic databases. The same PowerPoint presentation was used for both groups and the learning objectives were to:

1. Recall the scope and content of different bibliographic databases.
2. Select appropriate database(s) for researching a topic.
3. Recognize when to utilize different search options.
4. Demonstrate an effective database search in Ovid MEDLINE.
5. Identify components of a comprehensive and sensitive search approach.
6. Recognize that literature searching is an iterative process.
7. Manage search results and search histories.

The same demonstrations in Ovid MEDLINE were provided in-person and online and both instructional sessions were 80 minutes in length. Participants in the control group were able to ask questions throughout the session.

### Study design

At the end of the library orientation on September 13, 2022, students were given a Letter of Information and Consent for this study. Students were asked to complete and return the form before leaving class. The form indicated that students who do not agree to participate in the study would receive the standard in-person library session on literature searching. Students agreeing to participate in the study were randomly assigned to a control group (to receive in-person library instruction) or to an intervention group (to receive a narrated PowerPoint video). Consent forms where students agreed to participate were piled together and then shuffled (signature side down) before using the order of the stacked consent forms to assign students to the control group (odd numbers) or the intervention group (even numbers). Participants received an email on September 16, 2022, notifying them of what group they had been assigned to, and therefore whether they needed to show up in-person for the upcoming library session on literature searching. On September 27, 2022, the intervention group received an email with the narrated PowerPoint video file, and the control group attended in-person library instruction.

To compare the effectiveness of the two different instruction formats, participants were asked to complete one pre- and two post-tests, which contained the same 10 questions but arranged in different orders (Online Supplement, Appendix 1). To evaluate the perceived effectiveness of the library instruction received and preferences for learning literature searching skills, participants were asked to complete an evaluation survey (Online Supplement, Appendix 2). A graduate student who had taken already taken this course agreed to meet with two of the author investigators via Zoom to read over the test questions and evaluation survey. During and after this meeting, the test and survey were revised with more clear and concise language. Qualtrics online survey tool was used to deploy the tests and survey. The control group was given time to complete the pre-test at the beginning of the in-person library session, and the first post-test and evaluation survey directly after the session. The intervention group received the PowerPoint video file on the same day the control group received the in-person library instruction. Participants in the intervention group were instructed to complete the pre-test directly before watching the video, and the first post-test and evaluation survey directly after watching the video. However, participants in the intervention group were given the option of completing the online literature search training any time before the individual consultation with the librarian. A reminder email was sent to the intervention group on October 19, 2022, instructing them to watch the video (and complete the corresponding tests and survey) before meeting with the librarian by the end of October.

Supplement, Appendix 1

Supplement, Appendix 2

To assess learning retention, a second and final post-test was emailed to all participants on December 1, 2022. A reminder email was sent to all participants on January 3, 2023, instructing them to complete the second post-test by January 11, 2023.

Data collected were anonymized using a system for coded identification across submissions. To match the pre- and post-tests and evaluation surveys, each participant entered a unique identifier at the beginning of each test and the survey. Participants were repeatedly prompted to enter the first 2 letters of the street they grew up on, the first 2 letters of the street they currently live on, and the first 2 letters of the high school they attended.

### Analysis

Data were entered into an Excel file designed for the study, and imported into IBM SPSS (version 28.0 for Windows, Armonk, New York, 2022) for statistical analysis. Data were initially assessed descriptively using frequencies and percentages for the categorical data and means and standard deviations for the continuous data. We analyzed and compared the effectiveness of the different instruction formats using paired samples t-tests for the total scores (pre-test, post 1 and post 2) to look at pre and post-test changes. The McNemar test was used to assess paired binary (correct and incorrect) responses for the individual items. Change scores were also calculated between the time points, and independent samples t-tests were used to compare the change scores of the two groups. We assessed perceptions and preferences about library instruction formats pertaining to literature searching using Fisher’s Exact tests and Pearson chi-square tests to examine between-group differences, as well as independent samples t-test using the ordinal data. This permitted a more concise assessment of the data and allowed subtle differences to be more apparent. All p-values are 2-sided, a p-value of <0.05 was used as the cut-point for statistical significance, and we made no adjustment for multiple comparisons.

## Results

Thirteen of 17 students agreed to participate in the study, however, one of these students was not available to receive in-person library instruction on the scheduled date. Since they could not be randomly assigned to one of the groups, they were not included in the study and instead received the narrated PowerPoint video file. There were six participants in both the control and intervention groups.

### Effectiveness

The ability to analyze pre- and post-test scores was affected by missing data due to the low completion rates of the pre- and post-tests among the intervention group (Table 1). We first compared combined total test score changes to determine if library instruction (regardless of the method) increased the study participants’ understanding of literature searching in bibliographic databases. Paired samples T-Test for scores out of 10 show an increase from the pre-test to post-test 1 (up 2.89) and from pre-test to post-test 2 (up 2.50) (Table 2). The score from post-test 1 to post-test 2 declined slightly (down 0.55) as some knowledge was not retained (Table 2). The improvement in combined scores from pre-test to post-test 1 and pre-test to post-test 2 were statistically significant (p<0.05), but the change from post-test 1 to post-test 2 was not (Table 2).

**Table 1:** Pre- and post-test scores and completion rates among the in-person and video groups

Participant	Group	Test scores (out of 10)		
		Pre	Post 1	Post 2
1	In-person	4	10	8
2	In-person	5	9	7
3	In-person	7	7	7
4	In-person	5	8	7
5	In-person	3	8	9
6	In-person	5	7	7
7	Video	1	N/A	N/A
8	Video	3	4	N/A
9	Video	5	8	9
10	Video	N/A	4	N/A
11	Video	7	9	7
12	Video	N/A	10	10

**Table 2:** Paired samples T-test statistics for combined total test score changes

Paired Samples T-Test Statistics					
	Total scores	Mean (out of 10)*	N (out of 12)†	Sth. Deviation	p-value
**Pair 1**	Pre-test	4.89	9	1.45	.002
	Post-test 1	7.78	9	1.72	
**Pair 2**	Pre-test	5.13	8	1.36	.011
	Post-test 2	7.63	8	.92	
**Pair 3**	Post-test 1	8.44	9	1.13	.214
	Post-test 2	7.89	9	1.17	

* The mean value for the same test is not the same across different pairs because participant test scores in each pair was based on responses available for the tests compared. †The number of participants in each pair is not the same because it was based on participant responses available for the tests compared.

We calculated the change between time points in pre- and post-test scores for each group and then compared the change between the two groups to account for knowledge scores at baseline. The findings show that the in-person group improved more than the video group from pre- to post-test 1 (up 1.3 more), and from pre- to post-test 2 (up 0.7 more), but the difference was not statistically significant (Table 3). Learning retention was measured by comparing post-test 1 and post-test 2 score changes by group. Scores in both groups dropped from post-test 1 to post-test 2 but the difference was not statistically significant (Table 3).

**Table 3:** Comparison of pre- and post-test score changes by group

Change in time points		N	Mean	Std. Deviation	p-value
Pre and post-test 1	In-person	6	3.3	2.2	.355
	Video	3	2.0	1.0	
Pre and post-test 2	In-person	6	2.7	2.1	.725
	Video	2	2.0	2.8	
Post 2 minus post 1	In-person	6	-.7	1.2	.729
	Video	3	-.3	1.5	

### Perceptions

All 12 study participants completed the online survey about their perceptions and preferences of library instruction methods for teaching literature searching. When asked “*How would you rate the pace of the training session?*”, all 6 participants in the video group responded with “*Just the right pace*”. Of the 6 participants in the in-person group, 4 responded with “*Just the right pace*”, 1 “*A little too fast*”, and 1 “*A little too slow*.” No respondents from either group responded with “*Much too fast*” or “*Much too slow*.” The Fisher’s Exact test p-value between groups for training session pace was not statistically significant (p=0.455).

When asked “*How clear was the following content presented*” for subtopics of the training session, the video group responded more favourably 6 times, in comparison to the in-person group responding more favorably 2 times; both groups had the same score 2 times (Table 4).

**Table 4:** Comparison of how clear the content was presented by group*

	Group	Mean	Std. Dev.	p-value
Scope and content of different bibliographic databases	In-person	3.50	.548	.260
	Video	3.83	.408	
When to utilize different search options (i.e. basic search, advanced search, subject headings)	In-person	3.17	.408	.549
	Video	3.33	.516	
Combining search terms using AND/OR	In-person	3.33	.816	.687
	Video	3.50	.548	
How to search with subject headings	In-person	3.17	.753	.209
	Video	3.67	.516	
Exploding subject headings	In-person	3.50	.548	1.000
	Video	3.50	.548	
Applying subheadings to subject headings	In-person	3.50	.548	1.000
	Video	3.50	.548	
Limiting search results by publication type, year, language etc.	In-person	3.83	.408	.549
	Video	3.67	.516	
Methods for a comprehensive search approach	In-person	3.33	.816	.687
	Video	3.50	.548	
Managing your search results	In-person	3.83	.408	.549
	Video	3.67	.516	
Saving and sharing your search history	In-person	3.33	.816	.418
	Video	3.67	.516	

* Likert scale scores: 1 Mostly unclear, 2 Somewhat unclear, 3 Somewhat clear, 4 Mostly clear

When asked “*How effective did you find this training session?*”, 4 participants in the video group responded with “*Mostly effective*”, and 2 with “*Somewhat effective*.” In comparison, 3 participants in the in-person group responded with “*Mostly effective*”, and 3 with “*Somewhat effective*.” No participants responded with “*Somewhat ineffective*” or “*Mostly ineffective*.” The Fisher’s Exact test p-value between groups for the perceived effectiveness of the training session suggested that the two groups were equivalent (p=1.00).

When asked “*How confident are you about performing the following tasks after receiving library instruction*” for subtopics of the training session, the video group responded more favourably 6 times, in comparison to the in-person group responding more favorably 3 times; both groups had the same score once (Table 5). However, when asked about overall confidence with literature searching before and after receiving library instruction, the in-person group were slightly more confident than the video group afterwards, but they reported being more confident beforehand (Table 6).

**Table 5:** Comparison of confidence performing literature searching tasks after receiving library instruction by group*

	Group	Mean	Std. Dev.	p-value
Choosing relevant bibliographic databases for literature searches	In-person	3.83	.408	.086
	Video	3.17	.753	
Utilizing basic search	In-person	3.67	.516	1.000
	Video	3.67	.516	
Combining search terms using AND/OR	In-person	3.33	.816	.418
	Video	3.67	.516	
Searching with subject headings	In-person	3.33	.516	.599
	Video	3.50	.548	
Exploding subject headings	In-person	3.83	.408	.549
	Video	3.67	.516	
Applying subheadings to subject headings	In-person	3.00	.632	.174
	Video	3.50	.548	
Limiting search results by publication type, year, language etc.	In-person	4.00	.000	.145
	Video	3.67	.516	
Utilizing a comprehensive search approach	In-person	3.00	.000	.049
	Video	3.50	.548	
Managing your search results	In-person	3.00	.894	.145
	Video	3.67	.516	
Saving and sharing your search history	In-person	3.17	.753	.209
	Video	3.67	.516	

* Likert scale scores: 1 Mostly unsure, 2 Somewhat unsure, 3 Somewhat confident, 4 Mostly confident

**Table 6:** Comparison of confidence with literature searching before and after receiving library instruction by group

How would you rate your confidence with literature searching in bibliographic databases before and after this session?	In-person		Video	
	Before	After	Before	After
Mostly unsure	1		1	
Somewhat unsure	2		4	
Somewhat confident	2	3	1	4
Mostly confident	1	3	0	2
Total	6	6	6	6

### Preferences

**Fig. 1:**
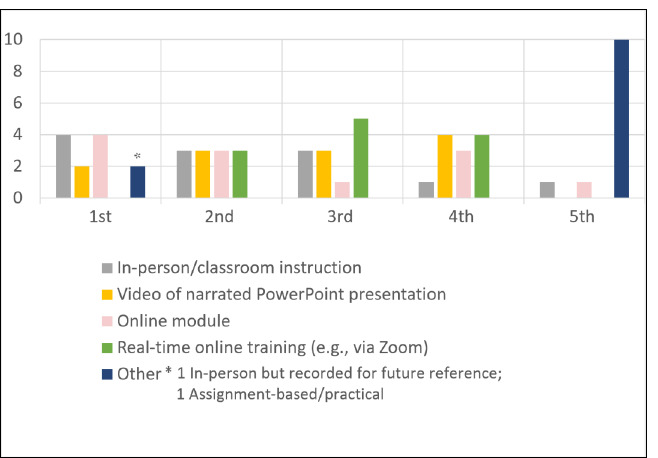
Preferred instruction methods for learning about literature searching in bibliographic databases (n=12)

**Fig. 2:**
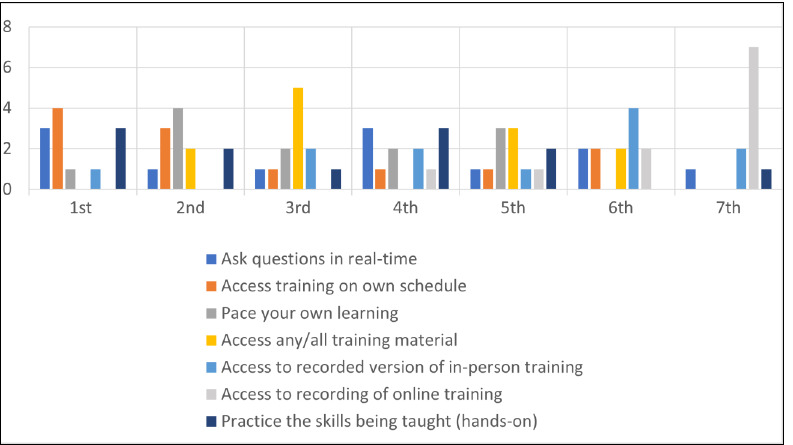
Factors most important for receiving instruction about literature searching in bibliographic databases (n=12)

The online survey asked study participants to rank their preferred instructional method for learning about literature searching in bibliographic databases in order. Asynchronous online library instruction was the number 1 answer for 6 participants (4 for online module and 2 for narrated PowerPoint video), followed by inperson/classroom instruction for 5 participants (Figure 1). Real-time online instruction (e.g., via Zoom) was the least preferred option for literature search instruction [9,13–17] (Figure 1). The online survey also asked participants to rank the factors they consider most important for receiving instruction about literature searching in bibliographic databases. Participants valued being able to access training on their own schedule most of all, followed by the ability to practice the skills being taught (hands-on training), and the ability to pace their own learning (Figure 2).

Participants ranked the ability to access recorded versions of in-person and online training as least important (Figure 2).

## Discussion

### Effectiveness

These findings are similar to previous comparative studies where both in-person and online instruction were found to be effective for delivering information literacy content to post-secondary students, and where the difference in student performance was not statistically significant between in-person and online instruction groups [9,13–18]. There is, however, research evidence that online instruction was significantly more effective than in-person instruction for undergraduate students at a private university [19].

Although the difference was not significant, learning retention in the short term was slightly better for asynchronous online learners, where the time elapsed between library instruction and testing may have been shorter, depending on when students in the intervention group viewed the instructional video. Asynchronous online instruction can offer students more flexibility to learn, practice, and apply skills at the time-of-need than in-person and online synchronous instruction. For this reason, asynchronous online instruction may result in better learning assessment outcomes for students when completing assignments or tests.

### Perceptions

A study by Holman, which compared an online library tutorial to traditional classroom instruction for bibliographic instruction, found the perceived effectiveness of instruction methods to be very similar [13] In our study, the asynchronous online group rated the perceived effectiveness and their confidence to perform specific search tasks more favourably than the in-person group. However, like Hess’ study comparing face-to-face instruction, online instruction, or both methods for research skills including literature searching, there was not a significant difference in the perceived effectiveness of in-person and asynchronous online instruction in our study [18]. The asynchronous online instruction group in our study also rated the pace of library instruction more favourably than the in-person group (although the difference was not statistically significant), as they did in Holman’s study [13]. Like Tomaszewski’s study in an undergraduate population, which compared in-person library instruction to video modules on topics such as search strategies in databases, students in the in-person group of our study were divided on pace, rating the session as either too fast, too slow, or just right [10]. In comparison, students in the asynchronous online session of our study all reported that the pace was just right. A valuable advantage of asynchronous online instruction over both in-person classroom instruction and synchronous online instruction is the ability for students to self-pace by pausing or going back to read or hear something again, if needed. Participants in our study were able to open the MP4 file in the video player of their choice and may have been able to further improve their ability to self-pace if they used a player that allows users to control the speed of the video. Unlike Holman’s study, students who received asynchronous online instruction in this study rated the clarity of learning specific search tasks more favourably than the in-person group; although, the difference was only statistically significant for one task [13]. This may speak to the difference in clarity that instructional videos with demonstrations (that can be paused and replayed) can provide over synchronous instruction and text-based online tutorials. Participants in the control group of this study were provided with the PowerPoint slides presented in-person after the session, but these materials may be of limited value without narration and demonstrations to explain and visualize the content. Although it was not possible to track how many times individual participants accessed the video file in our study, this data would be insightful for asynchronous online instructors to collect and review, if the technology utilized permits such tracking.

### Preferences

Preferred instructional methods for learning about literature searching were slightly in favour of asynchronous online instruction (i.e. online modules or videos) over in-person classroom instruction in this study. The graduate students in this study valued being able to access training on their own schedule the most, which helps explain why synchronous online instruction (e.g. via Zoom) was the least preferred instruction method for learning about literature searching. Enhanced accessibility of asynchronous online instruction may also be a factor. Platforms like PowerPoint enable closed captioning and the option to create audio transcripts of recordings which can benefit students with hearing impairments and those whose first language is not English.

Our survey findings in the graduate student population are similar to those of Gibbs et al., who found that graduate students in the humanities and social sciences at Georgetown University have a preference for online instruction. Similarly, Hoffman et al. found a clear preference among graduate students in engineering, health sciences, medicine and dentistry, and science for online instruction in the form of online tutorials; although, in-person workshops were still found to be valuable [20,21]. Previous surveys have also found undergraduate students in favour of online instruction over in-person instruction, including students in a psychology course at the University of South Florida Tampa Campus, and students in the speech-language-hearing sciences department at a private university [14,19]. Other studies, where in-person instruction may not be the overall preference, have found that many individual students still prefer in-person instruction, or are undecided. For example, Bussell et al. found differences among the levels of graduate students. Master's students favored videos, followed by websites, while doctoral students preferred websites first, followed by in-person workshops, and then videos [22]. They also found that synchronous online workshop was the least preferred method of instruction for both groups [22]. An early study from the late '90s found that undergraduate biology students at the University of California, Los Angeles, were split on their preferred mode of instruction, with 37% preferring computer-assisted instruction, 32% preferring in-person lecturing, and 31% being undecided [23]. Undergraduate freshmen students in an English course at the State University of New York indicated general neutrality for online or in-class instruction, similar to master’s and doctoral students at the University of Otago, who showed no clear preference for online or face-to-face support [16,24].

Smith et al. found that instruction delivery methods need to be diverse because postgraduates’ needs are diverse. A combination of student, individual, and circumstantial factors may influence student preferences for library instruction:

- degree level
- discipline of study
- content type
- related learning assessment(s)
- motivation to learn the specific content
- preferred learning style
- familiarity with methods for delivering instruction (e.g. Zoom)
- disabilities
- time of day or year

For this reason, it may be almost impossible to please every student when deciding on an instruction method. As Holman states “There will always be students who thrive or fail in various types of instruction” [13]. As technology for delivering online instruction continues to evolve and improve, student preferences may shift as well. Some anticipate that the preference for asynchronous one-shot library workshops will only increase with incoming Generation Z students and beyond since videos have always been a commonly used resource for information and knowledge with this demographic cohort [10].

### Efficiency

While this study investigated the effectiveness of in-person and asynchronous online instruction methods, and evaluated student perceptions and preferences there within, library instructors should also take efficiency into consideration when deciding on an instruction method for delivering information literacy content. The librarian chose a narrated PowerPoint video for delivering the library session during the pandemic because the liaison librarian had already created one when another faculty member had asked them to develop an asynchronous online session for medical residents on the same topic. The librarian had already invested a significant amount of time to develop the narrated PowerPoint video and wanted to maximize its use. The librarian initially chose PowerPoint for making a video because they were already familiar with this method. Other methods for creating a video, like Camtasia, may be more user-friendly and efficient but would have involved learning a new software. Updating the PowerPoint slides, audio recordings for selected slides, and demonstrations to customize the video for the purpose of this study ended up taking much longer than expected. Hahn describes challenges of creating online video lectures in 2011 that are still relevant today:

One challenge was devoting the time needed to record and edit the video lectures. This included writing the scripts, recording and rerecording where necessary, and editing and enhancing each of the videos. There was also the additional challenge of being comfortable and accepting one’s voice and the overall outcome of the recordings. Overall, even the shortest and simplest videos required at least a couple of hours to produce, while those with a large number of captions and highlights took considerably longer [25].

In retrospect, it is plain to see that delivering the content online in real-time during the pandemic would have taken far less time than adapting a previously narrated PowerPoint. Synchronous online instruction would not only have been more efficient during the pandemic in this case, it also would have been relatively easy to coordinate because the students already had assigned class time allotted for the library session. The conclusion from a study published three decades ago, that asynchronous online library instruction is too labour-intensive to be cost-effective, may still be the case for many academic libraries today [23]. However, in cases where asynchronous online instruction can be used multiple times without the need for major revisions and updates, it may be more efficient than providing multiple instances of synchronous instruction. While there is a lack of library research that evaluates the effectiveness of synchronous instruction provided online, many of us are probably personally familiar with “Zoom fatigue” by now [26,27]. Other challenges to delivering synchronous online instruction via videoconferencing include a diminished educational experience reported by instructors and students resulting from low camera use [28]. A survey to better understand why students in higher education did not turn on their cameras during synchronous class sessions found that deterrents include concerns about their personal appearance, the physical location being shown in the background, and having a weak internet connection [28].

### Study limitations

Strengths of this study include randomizing students to a control and intervention group, but it is not without limitations. The four students who did not consent to participate in this study may have declined because of a preference to receive traditional in-person instruction; in which case, the preferences for learning about literature searching reported by study participants may not accurately reflect the preferences of all students enrolled in the course. The sample size for this study was small, making it difficult for differences to attain statistical significance. These findings may not be generalizable to other health sciences graduate students receiving library instruction for literature searching for various reasons. For example, the information literacy session in this comparative study was not a standalone session but part of a larger information literacy program for students taking this course. Benefits of traditional in-person instruction include the ability for students and the librarian instructor to meet face-to-face. All students in this study met the librarian instructor in-person for an initial information literacy session that introduced library resources and services. While practice time was not part of the information literacy session on literature searching, students were required to practice these skills before a mandatory follow-up meeting with the librarian to discuss the search strategy for their research question. During this oneon-one consultation with a librarian, students were able to receive feedback on their search approaches. Study participants may have been more receptive and positive about online instruction for literature searching in this study because of these factors , which were not considered as part of this study’s objectives.

### Future research

Future research into the effectiveness of different library instruction methods for graduate students in the health sciences (using a larger sample population to ascertain statistical significance) would help address a gap in the literature. Since these findings did not discover a correlation between learner preferences for information literacy instruction and improved test scores, this could be another interesting area for future research projects to investigate. Furthermore, most of the research to date focuses on comparing in-person instruction to asynchronous methods. Studies into synchronous online instruction methods such as teaching via Zoom or MS Teams are needed.

### Conclusions

This study compared in-person classroom instruction with asynchronous online instruction in the form of an online video for teaching translational medicine graduate students about literature searching in bibliographic databases. Like previous comparative studies have found, both in-person and asynchronous online instruction were effective for teaching information literacy content to students in higher education. While the sample size for this study was small, making it difficult to attain statistical significance, study participant responses were more often in favour of asynchronous online learning despite the in-person group improving more from pre-tests to post-tests. In contrast to synchronous instruction online and in-person, asynchronous online instruction offers students the flexibility of learning at the time of need and the ability to self-pace and replay the content as needed. Delivering information literacy content in the form of online videos may be considerably more time-intensive compared to synchronous instruction delivered in-person or online.

## Data Availability

The datasets generated and analyzed during the current study are openly available in the Borealis repository, licensed under a Creative Commons Attribution-ShareAlike 4.0 International License. They are available here: https://doi.org/10.5683/SP3/HW2TBA.

## References

[1] Shin N, Pine S, Martin C, Bardyn T. Academic library instruction in the time of a COVID-19 pandemic–lessons learned. J Web Librariansh [Internet]. 2021 Dec 19;16(1):1-30. Available from: 10.1080/19322909.2021.2015046.

[2] Cattaneo KH. Telling active learning pedagogies apart: from theory to practice. J New Approach Educat Res [Internet]. 2017 Jul 15;6(2). Available from: 10.7821/naer.2017.7.237.

[3] Zeng H, Luo J. Effectiveness of synchronous and asynchronous online learning: a meta-analysis. Interact Learn Environ [Internet]. 2023 Apr 25;32(8):4297-4313. Available from: 10.1080/10494820.2023.2197953.

[4] Jacques S, Ouahabi A, Lequeu T. Synchronous e-learning in higher education during the COVID-19 pandemic. In: IEEE Global Engineering Education Conference (EDUCON) [Internet]. IEEE Global Engineering Education Conference (EDUCON); 2021 Apr 21-23; Vienna, Austria[place unknown]: IEEE; 2021 Jun 18. 1102-9 p. Available from: 10.1109/EDUCON46332.2021.9453887.

[5] Pickard E, Sterling S. Information literacy instruction in asynchronous online courses: which approaches work best? Coll Res Libr [Internet]. 2022 Mar 3;83(2):184. Available from: 10.5860/crl.83.2.184.

[6] Lynn VA, Bose A, Boehmer SJ. Librarian instruction-delivery modality preferences for professional continuing education. J Med Libr Assoc [Internet]. 2010 Jan [cited 2022 Sep 14];98(1):57-64. Available from: 10.3163/1536-5050.98.1.017. PMC2801962 20098656

[7] Loo JL, Eifler D, Smith E, Pendse L, He J, Sholinbeck M, et al. Flipped instruction for information literacy: five instructional cases of academic librarians. J Acad Librariansh [Internet]. 2016 May 3;42(3): 273-80. Available from: 10.1016/j.acalib.2016.03.001.

[8] He J. Construction of “three-stage asynchronous” instructional mode of blended flipped classroom based on mobile learning platform. Educ Inf Technol [Internet]. 2020 May 9;25(6):4915-36. Available from: 10.1007/s10639-020-10200-9.

[9] Silk KJ, Perrault EK, Ladenson S, Nazione SA. The effectiveness of online versus in-person library instruction on finding empirical communication research. J Acad Librariansh [Internet]. 2015 Mar 1;41(2):149–54. Available from: 10.1016/j.acalib.2014.12.007.

[10] Tomaszewski R. A STEM e-class in action: a case study for asynchronous one-shot library instruction. J Acad Librariansh [Internet]. 2021 Sep 1;47(5):102414. Available from: 10.1016/j.acalib.2021.102414.

[11] Rehman R, Fatima SS. An innovation in flipped class room: a teaching model to facilitate synchronous and asynchronous learning during a pandemic. Pak J Med Sci [Internet]. 2021;37(1):131–6. Available from: 10.12669/pjms.37.1.3096. 33437264 PMC7794122

[12] Harkins MJ, Rodrigues DB, Orlov S. ‘Where to start?’: Considerations for faculty and librarians in delivering information literacy instruction for graduate students. Practica Acad Librariansh [Internet]. 2011 May 26 [cited 2024 Mar 17];1(1):28–50. Available from: https://pal-ojstamu.tdl.org/pal/article/view/1463.

[13] Holman L. A comparison of computer-assisted instruction and classroom bibliographic instruction. Ref Serv Q [Internet]. 2000 Sep;40(1):53–60. Available from: http://www.jstor.org/stable/20863900.

[14] Silver SL, Nickel LT. Are online tutorials effective? A comparison of online and classroom library instruction methods. Res Strategi [Internet]. 2005 Jan 1;20(4):389–96. Available from: 10.1016/j.resstr.2006.12.012.

[15] Germain CA, Jacobson TE, Kaczor SA. A comparison of the effectiveness of presentation formats for instruction: teaching first-year students. Coll Res Libr [Internet]. 2000 Jan;61(1):65-72. Available from: 10.5860/crl.61.1.65.

[16] Nichols J, Shaffer B, Shockey K. Changing the face of instruction: is online or in-class more effective? Coll Res Libr [Internet]. 2003 Sep;64(5):378-88. Available from: 10.5860/crl.64.5.378.

[17] Churkovich M, Oughtred C. Can an online tutorial pass the test for library instruction? An evaluation and comparison of library skills instruction methods for first year students at Deakin University. Aust Acad Res Libr [Internet]. 2002 Jan 1 [cited 2024 Apr 28];33(1):25–38. Available from: https://www.tandfonline.com/doi/abs/10.1080/00048623.2002.10755177.

[18] Hess AN. Online and face-to-face library instruction: assessing the impact on upper-level sociology undergraduates. Behav Soc Sci Librar [Internet]. 2014 Aug 11;33(3):132-147. Available from: 10.1080/01639269.2014.934122.

[19] Gorman EF, Staley C. Mortal or Moodle? A comparison of in-person vs. online information literacy instruction. J Lib Info Serv Dist Learn [Internet]. 2018 Aug 17;12(3–4):219-36. Available from: 10.1080/1533290X.2018.1498635.

[20] Gibbs D, Boettcher J, Hollingsworth J, Slania H. Assessing the research needs of graduate students at Georgetown University. J Acad Librariansh [Internet]. 2012 Sep;38(5):268-76. Available from: 10.1016/j.acalib.2012.07.002.

[21] Hoffmann K, Antwi-Nsiah F, Feng V, Stanley M. Library research skills: a needs assessment for graduate student workshops. Iss Sci Tech Librariansh [Internet]. 2008 May 1;(53). Available from: 10.29173/istl2440.

[22] Bussell H, Hagman J, Guder CS. Research needs and learning format preferences of graduate students at a large public university: an exploratory study. Coll Res Libr [Internet]. 2017;78(7):978. Available from: 10.5860/crl.78.7.978.

[23] Kaplowitz J, Contini J. Computer-assisted instruction: is it an option for bibliographic instruction in large undergraduate survey classes? Coll Res Libr [Internet]. 1998;59(1):19–27. Available from: 10.5860/crl.59.1.19.

[24] Smith SA, Lubcke A, Alexander D, Thompson K, Ballard C, Glasgow F. Listening and learning: myths and misperceptions about postgraduate students and library support. Ref Serv Rev [Internet]. 2019 Aug 13;47(4):594-608. Available from: 10.1108/RSR-03-2019-0019.

[25] Hahn E. Video lectures help enhance online information literacy course. Ref Serv Rev [Internet]. 2012 Feb 10;40(1):49–60. Available from: 10.1108/00907321211203621.

[26] Epstein HAB. Virtual meeting fatigue. J Hosp Librariansh [Internet]. 2020 Oct 7;20(4):356-60. Available from: 10.1080/15323269.2020.1819758.

[27] Nesher Shoshan H, Wehrt W. Understanding “Zoom fatigue”: a mixed-method approach. App Psych [Internet]. 2022 Jun 20;71(3):827-52. Available from: 10.1111/apps.12360.

[28] Castelli FR, Sarvary MA. Why students do not turn on their video cameras during online classes and an equitable and inclusive plan to encourage them to do so. Ecol Evol [Internet]. 2021 Jan 10;11(8). Available from: 10.1002/ece3.7123. PMC8057329 33898009

