# Supplementary material for: Comparison of asynchronous online versus in-person library instruction methods for teaching literature searching to graduate students
Source: J Can Health Libr Assoc. 2024 Dec 1;45(3):147–60. doi: 10.29173/jchla29792 (PMC11881644; doi:10.29173/jchla29792)
Supplement: Supplementary file 1 [file JCHLA-45-147-s001.pdf]

Appendix 1

TMED 802 Pre-Test: Literature Searching in Bibliographic Databases

In order to match pre- and post-tests and the evaluation survey, we will be prompting you to provide a unique but anonymous identifier. For the six-digit identifier, please use the first 2 letters of the street you grew up on, followed by the first 2 letters of the street you currently live on, followed by the first 2 letters of the high school you attended, **all in capital letters**. For example, I grew up on Old Madoc Road, currently live on Boxwood Street, and went to Centennial Secondary School. My identifier would be: OLBOCE.

Please enter your unique identifier: _ _ _ _ _ _

1. Performing a literature search in the relevant bibliographic database(s) can help:
   a) Review the research landscape surrounding your topic
   b) Ascertain whether a research question has already been answered by other researchers
   c) Investigate how similar projects were conducted
   d) Identify eligible studies for systematic reviews
   e) All of the above

2. Which of the following statements about using the basic search box in Ovid MEDLINE is FALSE?
   a) The basic search box is a quick and easy way to locate some sources on a topic
   b) The basic search algorithm attempts to include related terms for your entered search terms
   c) All search terms are automatically combined with AND
   d) Search terms cannot be 'OR-ed' in the basic search box
   e) The basic search box can be used for a comprehensive search

3. Which of the following statements about database subject headings is FALSE?
   a) Subject headings are applied to identify the main concepts of a bibliographic record
   b) MEDLINE subject headings are referred to as MeSH (Medical Subject Headings)
   c) All bibliographic records in Ovid MEDLINE are indexed with subject headings
   d) Embase and MEDLINE use different subject heading systems
   e) Subject headings are organized into hierarchical relationships of broader and narrower terms

4. "Exploding" a subject heading in databases on the Ovid platform will search for bibliographic records with:
   a) The selected subject heading only
   b) The selected subject heading and any narrower subject headings in the hierarchy
   c) The selected subject heading and the broader subject heading in the hierarchy
   d) The selected subject heading and all subheadings

e) The selected subject heading without any subheadings

5. When searching Ovid MEDLINE, which of the following strategies will DECREASE the number of search results?
   a) Exploding a subject heading
   b) Applying a subheading such as "therapeutic use" to a subject heading
   c) Combining search terms using OR
   d) Truncating keywords (a.k.a. text words) to retrieve variant word endings
   e) All of the above

6. When searching Ovid MEDLINE, which of the following strategies will INCREASE the number of search results?
   a) **Exploding a subject heading**
   b) Focusing a subject heading
   c) Applying a subheading such as "therapeutic use" to a subject heading
   d) Combining search terms using AND
   e) Applying limits for publication type, date, or language

7. Which is the best way of truncating the following keyword (a.k.a. text word) search to retrieve multiple word endings such as intestine, intestines, intestinal, intestinally, etc. in Ovid MEDLINE advanced search:
   a) intestine*
   b) intestin+
   c) intestin*
   d) intestin#
   e) intestine+

8. Which of the following statements about bibliographic databases is FALSE?
   a) MEDLINE and Embase are the two main bibliographic databases for medicine
   b) MEDLINE and Embase allow you to search for terms within full text articles
   c) MEDLINE and Embase contain international content
   d) Embase contains bibliographic records for journal articles and conference abstracts
   e) MEDLINE can be searched via PubMed or Ovid MEDLINE

9. Searching multi-disciplinary databases can help identify potentially relevant sources that may be published outside of the health sciences, for example, an article about medical education that is published in an education journal rather than a medical journal. Which of the following is a multi-disciplinary database?
   a) PubMed
   b) Embase
   c) Web of Science
   d) PsycINFO
   e) BIOSIS Previews

10. Which of the following is generally NOT a characteristic of a COMPREHENSIVE literature search?
    a) Published and unpublished (“grey”) literature is searched
    b) A combination of subject heading and keyword (a.k.a. text word) searching is used
    c) Multiple databases are searched
    d) Search results are limited to the most recent research
    e) Subject headings are exploded when appropriate
