# Supplementary material for: Comparison of asynchronous online versus in-person library instruction methods for teaching literature searching to graduate students
Source: J Can Health Libr Assoc. 2024 Dec 1;45(3):147–60. doi: 10.29173/jchla29792 (PMC11881644; doi:10.29173/jchla29792)
Supplement: Supplementary file 2 [file JCHLA-45-147-s002.pdf]

Appendix 2

Evaluation of TMED 802 library session and attitudes about library instruction methods

Start of Block: Default Question Block

Instructions Dear TMED 802 graduate students,

Please consider the library training session on *Literature Searching in Bibliographic Databases* when answering the following questions. We estimate that this questionnaire will take around 5-10 minutes to complete. Your thoughtful participation is greatly appreciated and will contribute to the improvement of library instruction and student learning. Your responses will be kept completely anonymous and will not be attached to personal identifiers.

In order to match pre- and post-tests and the evaluation survey, we will be prompting you to provide a unique but anonymous identifier. For the six-digit identifier, please use the first 2 letters of the street you grew up on, followed by the first 2 letters of the street you currently live on, followed by the first 2 letters of the high school you attended, all in capital letters.

For example, I grew up on Old Madoc Road, currently live on Boxwood Street, and went to Centennial Secondary School. My identifier would be: OLBOCE.

Unique Identifier Please enter your unique identifier

____

Q1. How old are you?

- ○ 21 and under (1)
- ○ 22-25 (2)
- ○ 26-29 (3)
- ○ 30-33 (4)
- ○ 34 and over (5)

Q2. What is the highest academic degree that you have already completed?

- ○ Bachelor’s degree (1)
- ○ Master's degree (2)
- ○ PhD (3)
- ○ MD (4)
- ○ MD and Master's degree (5)
- ○ MD and PhD (6)
- ○ Post-doctorate (7)

Q3. How would you rate your confidence with literature searching in bibliographic databases *before* this training session?

- ◯ Mostly unsure (1)
- ◯ Somewhat unsure (2)
- ◯ Somewhat confident (3)
- ◯ Mostly confident (4)

Q4. What format of instruction did you receive for this training session?

- ◯ In-person/classroom instruction (1)
- ◯ Video of narrated PowerPoint presentation (2)

Q5. How would you rate the pace of the training session?

- ◯ Much too fast (1)
- ◯ A little fast (2)
- ◯ Just the right pace (3)
- ◯ A little too slow (4)
- ◯ Much too slow (5)

Q6. How clear was the following content presented?

	Mostly unclear	Somewhat unclear	Somewhat clear	Mostly clear
	1	2	3	4

Scope and content of different bibliographic databases ()	
When to utilize different search options (i.e.,basic search, advanced search, subject headings) ()	
Combining search terms using AND/OR ()	
Value of searching with subject headings ()	
How to search with subject headings ()	
Exploding subject headings ()	
Applying subheadings to subject headings ()	
Limiting search results by publication type, year, language etc. ()	
Methods for a comprehensive search approach ()	
Managing your search results ()	
Saving and sharing your search history ()	

Q7. How confident are you about performing the following tasks in bibliographic databases after this training session?

	Mostly unsure	Somewhat unsure	Somewhat confident	Mostly confident
	1	2	3	4

Choosing relevant bibliographic databases for literature searches ()	
Utilizing basic search ()	
Combining search terms using AND/OR ()	
Searching with subject headings ()	
Exploding subject headings ()	
Applying subheadings to subject headings ()	
Limiting search results by publication type, year, language etc. ()	
Utilizing a comprehensive search approach ()	
Managing your search results ()	
Saving and sharing your search history ()	

Q8. Overall, how would you rate your confidence with literature searching in bibliographic databases *after* this training session?

- Mostly unsure (1)
- Somewhat unsure (2)
- Somewhat sure (3)
- Mostly sure (4)

Q9. How effective did you find this training session?

- ○ Mostly ineffective (1)
- ○ Somewhat ineffective (2)
- ○ Somewhat effective (3)
- ○ Mostly effective (4)

Page Break

Q10. Please rank your preferred instructional method for learning about literature searching in bibliographic databases in order (using drag and drop; 1 being most preferred):

______ In-person/classroom instruction (1)

______ Video of narrated PowerPoint presentation (2)

______ Online module (3)

______ Real-time online training (e.g., via Zoom) (4)

______ Other. Please specify: (5)

Q11. Please rank the factors you consider most important for receiving instruction about literature searching in bibliographic databases (using drag and drop; 1 being most important):

______ Ability to ask questions in real-time (1)

______ Ability to access training on your own schedule (e.g., at the point of need) (2)

______ Ability to pace your own learning (e.g., using pause/stop with video recordings) (3)

______ Ability to access any/all training materials (e.g., PowerPoint slides) (4)

______ Ability to access a recorded version of in-person training (5)

______ Ability to access a recorded version of online training (e.g., Zoom recording) (6)

______ Ability to maintain privacy when training is recorded (e.g., not having Q&A portion recorded) (7)

______ Ability to practice the skills being taught (hands-on training) (8)

______ Other. Please specify: (9)

Q12. Is there anything else you would like to share with us about this training session or your learning preferences?

____

End of Block: Default Question Block
